# From genome-wide association studies to disease mechanisms: celiac disease as a model for autoimmune diseases

**DOI:** 10.1007/s00281-012-0312-1

**Published:** 2012-05-14

**Authors:** Vinod Kumar, Cisca Wijmenga, Sebo Withoff

**Affiliations:** Department of Genetics, University Medical Hospital Groningen, University of Groningen, PO Box 30001, 9700 RB Groningen, the Netherlands

**Keywords:** Celiac disease, Autoimmune disease, Immune-related disease, Genome-wide association studies, GWAS, Pathway analysis

## Abstract

Celiac disease is characterized by a chronic inflammatory reaction in the intestine and is triggered by gluten, a constituent derived from grains which is present in the common daily diet in the Western world. Despite decades of research, the mechanisms behind celiac disease etiology are still not fully understood, although it is clear that both genetic and environmental factors are involved. To improve the understanding of the disease, the genetic component has been extensively studied by genome-wide association studies. These have uncovered a wealth of information that still needs further investigation to clarify its importance. In this review, we summarize and discuss the results of the genetic studies in celiac disease, focusing on the “non-HLA” genes. We also present novel approaches to identifying the causal variants in complex susceptibility loci and disease mechanisms.

## Introduction

Immune-related diseases range from autoimmune diseases such as celiac disease (CD), rheumatoid arthritis (RA), multiple sclerosis (MS), and type I diabetes (T1D), to more chronic inflammatory disorders such as asthma and inflammatory bowel disease (IBD). Together these disorders now account for 5–10 % of all disease cases in Western countries (see elsewhere in this issue).

Celiac disease is one of the best-understood immune-related diseases. It is the most common food intolerance in humans, affecting at least 1 % of the Western population. It is a multifactorial disease caused by many different genetic factors that act in concert with non-genetic causes. A genetic association between CD and the HLA class II genes in the major histocompatibility complex (MHC) was documented almost 40 years ago [1]. One of the most important triggering factors is dietary gluten, a storage protein present in wheat and related grains (hordein in barley, secalin in rye, and avedin in oats) (see elsewhere in this issue).

CD is an excellent model for studying the contribution of genetic factors to immune-related disorders because: (1) the environmental triggering factor is known (gluten), (2) as in other autoimmune diseases, specific HLA types (*HLA-DQA1* and *HLA-DQB1* in the case of CD) are critically involved (see elsewhere in this issue), (3) there is involvement of non-HLA disease-susceptibility loci, many of which are shared with other autoimmune diseases, (4) there is an elevated incidence of other immune-related diseases both in family members and individuals, and (5) both the innate and the adaptive immune responses play a role in CD [2].

Prior to genome-wide association studies (GWAS) the genetics of CD included candidate gene studies in case-control cohorts and linkage studies in multi-generation families and affected sibpairs [3]. None of these studies have convincingly resulted in the identification of genetic factors beyond the well-established HLA-DQA1 and HLA-DQB1 genes. With the introduction of GWAS, the number of genetic factors implicated in CD has increased and 54 % of its heritability can now be explained. However, the methods for calculating the heritability are currently under debate [4], but CD remains the immune-related disorder with the best-characterized genetic component (e.g., MS 20 %, RA 16 %, CrD 23 %, UC 16 %, T1D 45 %) [5, 6].

## GWAS in CD: yielding only the tip of the iceberg

GWA studies provide an unbiased approach for identifying genes and pathways involved in a certain phenotype, as they are not based on prior biological knowledge of the genes that they identify. Indeed, GWAS frequently identify genes and/or pathways that were not previously implicated in the phenotype of interest, for example, the unexpected role of the autophagy pathway in IBD [7]). Such an unbiased approach is highly beneficial as it generates new hypotheses that open up new avenues for investigation. Nevertheless, we must be careful in interpreting GWAS findings, as it is sometimes difficult to pinpoint the primary target of the genetic association. It is important to realize that the gene names of disease-associated loci are merely signposts. Often it is difficult to identify the single gene or gene variant providing risk or protection to a disease, because disease-associated loci often contain multiple genes and potential risk variants. Since individual genetic risk variants are usually common and have only a modest effect on disease risk, and because the cell or a sample of the tissue where the disease manifests is difficult to obtain for research purposes, it is difficult to investigate the consequence of the true causal risk variant. Despite these hurdles, GWAS have uncovered hundreds of loci associated to immune-related disorders, although these may represent only the tip of the iceberg [8–10]. This wealth of information will serve to formulate hypotheses that can be tested using experimental studies. Moreover, GWAS data can also be subjected to bioinformatic analysis to obtain more details about the tip of the iceberg and to reveal what still remains under the surface (see later sections in this review). To appreciate the complexity of GWAS, it is important to fully grasp the statistics involved. The interested reader can find an extensive description of the analytical methods in a review by Balding [11]. Here, we will describe how GWAS have contributed to our understanding of the genetics of CD.

The first GWAS for CD was performed in 2007 on a relatively small cohort consisting of 778 CD patients and 1,422 controls, all from the UK [12]. The subjects were tested for association to some 300,000 genetic variants in the human genome (so-called single nucleotide polymorphisms or SNPs) and the top 1,500 most associated SNPs were followed-up in replication cohorts consisting of 1,643 cases and 3,406 controls. Besides HLA, 13 regions in the genome were identified as harboring genes and genetic variants associated to CD [12–14]. Interestingly, the majority of the identified regions contained genes controlling immune responses, such as the *IL2-IL21* locus on 4q27, thereby suggesting, for the first time, the potential role of IL2, a cytokine important for the homeostasis and function of T cells, and of IL21, a new member of the type 1 cytokine superfamily which regulates many other immune and non-immune cells. This first GWA study also revealed the phenomena of pleiotropy, i.e., genetic variants associated to CD are also associated with other immune-related diseases. For example, the *IL2-21* locus is now a well-established disease susceptibility locus for T1D, RA, UC, MS, and systemic lupus erythematosus (SLE) [2, 15–22].

A much larger GWAS on CD included more than 4,500 CD patients and nearly 11,000 controls from four different populations (UK, Italy, Finland, the Netherlands) and 550,000 SNPs [23]. After replicating the most-significant 131 SNPs in seven follow-up cohorts of European descent, comprising almost 5,000 CD patients and more than 5,500 controls, 13 new regions in the genome were found to be associated with CD, bringing the total number of non-HLA associated loci to 26. The study by Dubois et al. [23] also showed that about 50 % of CD-associated SNPs affect the expression of nearby genes (so-called expression quantitative traits loci or eQTLs), indicating that the mechanism underlying CD is governed by a deregulation of gene expression.

More recently, the number of loci associated to CD was raised to 39 [24] when the Immunochip platform became available [25] (see fine-mapping approaches).

The “resolution” of GWAS heavily depends on the number of samples included. One way to circumvent this limitation is to combine datasets and to perform a meta-analysis, as was done by Dubois et al. [23]. Given the pleiotropic nature of the genetics underlying immune-related diseases, it also became possible to conduct cross-disease meta-analyses aimed at identifying additional shared susceptibility loci, as has been successfully demonstrated for CD. Two published GWAS datasets, one on CD [23] and one on RA [16], were pooled and the data obtained from the primary analysis was replicated using 2,169 CD cases (and 2,255 controls) and 2,845 RA cases (and 4,944 controls). In this meta-analysis, eight SNPs were replicated, including four SNPs mapping to loci that had not previously been associated with either disease (*CD247*, *UBEL3*, *DDX6*, and *UBASH3A*) and another four SNPs mapping to loci that had previously only been established in one of the diseases (*SH2B3*, *8q24.2*, *STAT4*, and *TRAF1-C5*). The identification of these eight loci, together with six known loci (*MMEL1/TNFRSF14*, *REL*, *ICOS/CTLA4*, *IL2/IL21*, *TNFAIP3*, and *TAGAP*), brought the total number of non-HLA susceptibility loci shared between CD and RA to 14 [17]. A similar study was performed for CD and CrD and identified four shared susceptibility loci [21]. Although meta-analysis can help identify shared risk loci, it is important to realize that it is also possible to obtain contradictory data. Sometimes the association to the same loci is more complex and observed with different SNPs, or with identical SNPs but with the opposite allele. For example, the A allele of SNP rs917997 in *IL18RAP* is increased in frequency in CD cases, while the same allele is decreased in frequency in T1D patients [26]. This could mean that the SNP is protective in one disease and a risk factor in the other.

## Fine-mapping approaches

**Fig. 1:**
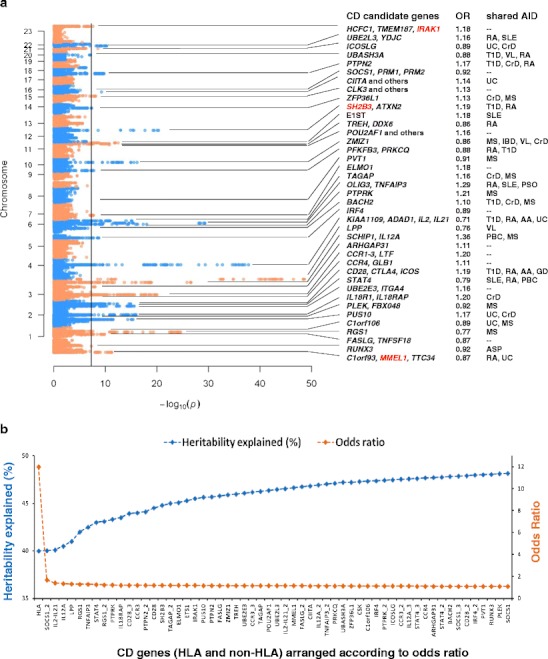
Overview of the celiac disease loci. **a** Manhattan plot showing the CD susceptibility loci identified by Immunochip. The *x*-axis displays the − log10 *P* values and the *y*-axis displays the chromosomes. Candidate genes from 39 loci are shown in the first text column. At three loci (*IRAK1, SH2B3*, and *MMEL1*), the most significant SNPs at each locus are in absolute linkage with coding variants. Next, the odds ratios (OR) of all CD SNPs are displayed. In the last column 28 CD loci are also shown to be susceptibility regions for other autoimmune diseases (the shared disease associations are extracted from the GWAS catalogue (www.genome.gov/gwastudies)). *AA* alopecia areata, *AID* autoimmune disease, *ASP* ankylosing spondylitis, *CrD* Crohn’s disease, *IBD* inflammatory bowel disease, *MS* multiple sclerosis, *PBC* primary biliary cirrhosis, *PSO* psoriasis, *RA* rheumatoid arthritis, *SLE* systemic lupus erythematosus, *T1D* type I diabetes, *UC* ulcerative colitis, *VL* vitiligo. **b** Odds ratios (OR) and cumulative heritability associated with each locus. Along the *x*-axis all the CD risk loci are arranged according to decreasing OR. Multiple independent signals at one locus are depicted as “gene name”_2 or “gene name”_3 (e.g., *SOCS1_1, SOCS1_2*, and *SOCS1_3* indicate three independent signals at the SOCS1 locus). We assumed a CD heritability of 89 % [75] and CD prevalence of 1.5 % to estimate the cumulative heritability explained. The OR of 12 for HLA [74] and the ORs of the non-HLA CD loci [24] were published previously

One of the problems associated with GWAS is that the genome is not necessarily covered at a high resolution. The early GWAS chips used in CD studies contained 300,000–550,000 SNPs, while the human genome consists of 3 billion basepairs, of which at least 1-2 % is polymorphic in any given individual. Many loci are therefore not covered densely enough with SNPs, resulting in the association with disease of regions that can contain multiple genes. This complicates the interpretation of the GWAS results, but one of the most straightforward approaches to address this problem is to fine-map disease-associated loci by zooming in on specific collections of SNPs that cover defined gene-sets at high density. A recent genetic study aimed at fine-mapping CD GWAS loci was performed on the Immunochip platform [24]. The Immunochip [25] is a custom Illumina Infinium HD array, which was specifically designed by the Immunochip Consortium to densely fine-map existing GWAS loci and to replicate loci that had not yet reached genome-wide significance. The approximately 200,000 SNPs on the Immunochip array consist of SNP variants that were present in public databases at the time of production (September 2009), including variants described in the European samples sequenced as part of the 1000 Genomes Project pilot phase I. The Immunochip covers: (1) the 186 loci associated with autoimmune or inflammatory diseases meeting genome-wide significance criteria (*P* < 5 × 10^−8^), from 12 immune-mediated diseases (autoimmune thyroid disease, ankylosing spondylitis, CD, CrD, IgA deficiency, MS, primary biliary cirrhosis, psoriasis, RA, SLE, T1D, and UC), (2) the MHC and KIR/LILR loci, (3) the most significant SNPs from GWAS loci with sub-significant *P* values awaiting deep replication, and (4) a small proportion of SNPs of investigator-specific undisclosed content. In the case of CD, the Immunochip was used to genotype more than 12,000 CD patients and a similar number of controls from seven different populations [24]. The platform revealed a total of 39 genome-wide significant loci (Fig. 1a), but upon conditional analysis 13 loci were found to include more than one independent association signal, resulting in a total of 57 independent non-HLA signals. These 57 SNPs are in general rather common, with frequencies above 5 % and modest effect sizes with an odds ratio between 1.124 and 1.360 (compared to an odds ratio of >5 for HLA) (Fig. 1b). Because of the higher density of SNPs for each of the loci, it was possible to refine the association signal to a single gene for 29 loci (Fig. 1a).

One of the most surprising findings from this fine-mapping study was the observation that the *PTPRK* gene is the causal gene in the *THEMIS*/*PTPRK* locus [23]. Immunological publications on the function of the *THEMIS* gene had suggested that it could be a very interesting candidate risk gene for CD, as it is an important regulator of thymic T cell selection [27]. This observation suggested an important role for the thymus; this is an attractive theory given the lack of oral tolerance present in CD. However, there is only limited literature on the *PTPRK* gene, but knock-out of the *Ptprk* gene in rats leads to a Th cell deficiency [28]. This example shows that GWAS results can easily be misinterpreted if attractive candidates are chosen without performing further validation. Immunochip analysis also identified 147 non-CD autoimmune disease loci with intermediate *p* values (in GWAS only SNPs with a *P* < 5 × 10^−8^ are considered true associations as they have reached “genome-wide significance”). It cannot be ruled out that these SNPs play a role in the disease process but that the study was underpowered to unequivocally prove involvement of these SNPs, suggesting that there might be dozens more genes contributing to CD.

Another approach for fine-mapping is imputation [29, 30]. Imputation is an in silico process in which the allelic combinations of non-genotyped SNPs in an individual are inferred (though not directly assayed) based on the haplotype structure present in large reference datasets, such as the ones provided by the 1000 Genomes Project (2010) and the International HapMap project [31–33]. A haplotype is the combination of alleles at adjacent locations (loci) on the chromosome that are transmitted together. After imputation, each dataset typically contains information on 2.5–4 million SNP variants per individual, including low-frequency variants that are not covered on a typical GWAS array [34]. Subsequent association analysis on imputed genotypes may narrow down the region of association and help pinpoint the causative variant. As imputation is merely an in silico prediction of unknown genotypes based on the haplotype structure of a reference population, sufficient quality control measures are needed to exclude badly imputed SNPs and then the predicted genotypes need to be validated by other genotyping techniques or direct sequencing.

## Genetic architecture of celiac disease

**Fig. 2:**
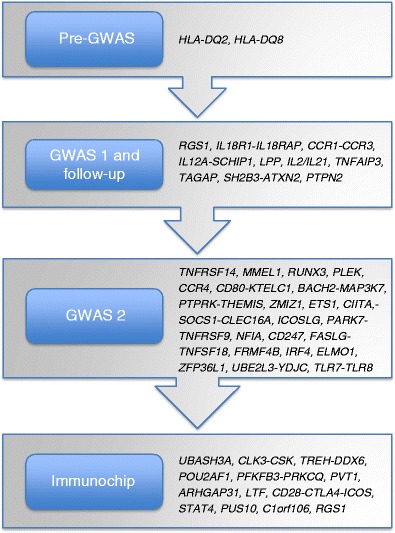
History of celiac disease genetics. The final Immunochip analysis increased the number of independent non-HLA CD susceptibility SNPs to 57 (see text for further details)

The studies conducted thus far (Fig. 2) suggest that the genetic architecture of CD follows the common disease-common variant (CD-CV) hypothesis [35–37]. To date, approximately 54 % of the genetics of CD can be explained by HLA plus the 57 non-HLA SNPs. The CD-CV hypothesis suggests that the remainder of the CD iceberg will also consist of common variants with very small effect sizes. Since identifying more of these variants would require extremely large cohort sizes, this would be very difficult to realize. There are several reasons why it is conceivable that the design of the current studies inhibits identification of less common genetic variants (with allele frequencies between 1–5 %): (1) the GWAS genotyping platforms are skewed towards covering common variants; (2) rare variants tend to be more population-specific but the studies conducted with the Immunochip for instance—which does contain low-frequency variants—did not take separate populations into account, thereby probably missing population-specific effects. The ultimate way to identify low frequency (allele frequency 1–5 %) and rare variants (allele frequency <1 %) requires different technologies, such as deep sequencing. The advent of whole genome sequencing is expected to reveal much of the landscape of rare variation [38], but for large population studies this approach is currently still too expensive. Another option is testing for the existence of rare variants with high effect sizes, but this requires a different strategy. Each population should be investigated separately for the disease-associated haplotype, which then needs to be resequenced to identify all the possible variants on it. However, the CD GWAS cohorts studied so far mostly consisted of populations of European descent, which limits the variation in predisposing haplotypes. To capture the vast majority of potential disease-causing rare variants would thus require as many different (multi-ethnic) CD cohorts as possible. Comparing haplotypes across different populations also has some additional advantages and may result in even more refinement of established association signals or help in identifying population-specific risk haplotypes/variants. For example, genotyping tag-SNPs at *TNFAIP3*, one of the autoimmune risk loci, in an African-American SLE cohort revealed a novel African-derived risk haplotype that was in linkage disequilibrium (LD) with a non-synonymous coding SNP [39], whereas in another study [40] re-sequencing of the same region in Europeans and Koreans revealed a deletion of T, followed by a T > A transversion in a non-coding region that showed much stronger odds ratio in Koreans than Europeans for SLE (odds ratio = 2.54 versus 1.7 in Europeans). Thus, the use of multi-ethnic disease cohorts for fine-mapping the disease-associated regions can be a powerful approach.

Now that a plethora of CD susceptibility factors has been identified, the challenge is to pinpoint the causal variants from each locus, and to prove that these causal variants affect the function of tissues and cell types involved in CD. Meeting this challenge requires a multidisciplinary approach, involving the generation and integration of bioinformatic, genetic, immunological and cell biological experimental data and clinical data. Below we will discuss the strategies that can be employed to meet this challenge, while focusing on the non-HLA CD susceptibility loci.

## Regulatory regions

**Fig. 3:**
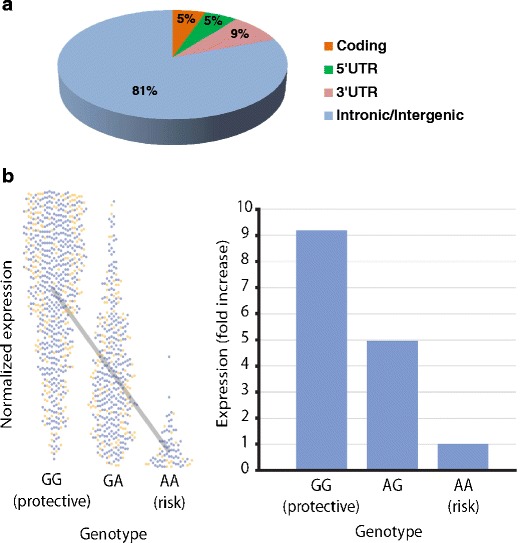
Location and effect of CD risk SNPs. **a** Genomic location of the SNPs. Proxy SNPs (*R* ^2^ > 0.8) for 57 CD top SNPs were extracted using the 1000 Genomes Project CEU population. Only three (5 %) of the 57 SNPs were in linkage with coding variants. About 5 and 9 % are located in the 5’-UTR and the 3’-UTR regions, respectively. This leaves 81 % of the variants to be located in non-coding regions of the genome (intergenic or intronic). The latter SNPs could be involved in the regulation of gene expression or they could affect non-coding RNA species. **b** Expression quantitative trait loci (eQTL) analysis at SNP rs917997. The figure shows the association of the risk genotype with a lower expression of *IL18RAP* (*P* = 1.1 × 10^−133^). The *left panel* displays the distribution of the normalized expression levels of *IL18RAP* mRNA according to the genotypes at rs917997. The *blue* and *orange dots* indicate samples from male and female volunteers, respectively. The *right panel* displays the foldchange in the levels of *IL18RAP* mRNA.

Until recently, the focus of genetic studies on autoimmune diseases has been on protein coding genes and many investigators expected to find SNPs that alter protein sequences, and thereby protein function. One of the most surprising findings from the recent study by Trynka et al. [24] is that only three of the 57 independent SNPs appear to affect protein sequences (in *MMEL1*, *SH2B3*, and *IRAK1*). A careful inspection of the finely mapped loci indicates that many SNPs map to either 5′ or 3′ untranslated regions (UTRs), to introns, or to intergenic regions (Fig. 3a). The *RUNX3*, *RGS1*, *ETS1*, *TAGAP*, and *ZFP36L1* genes show association with CD in the 5’-UTR region (i.e., 1st exon and 10 kb upstream of it), suggesting that the transcriptional regulation of these genes is affected by the CD-risk SNPs. There are different ways in which 5′-UTR SNPs can exert an effect on transcription, for example by altering or creating binding sites for transcription factors, or by modifying the binding sites for chromatin-modifying protein complexes, which in turn can affect DNA methylation and/or histone modification (‘epigenetic effects’) [41, 42]. The association of CD to *IRF4*, *PTPRK*, and *ICOSLG* seems to affect 3′-UTR sequences which, theoretically, could lead to a decrease in stability or increased degradation of the respective mRNAs, or to inhibition of translation by, for example, altering binding sites for RNA-stabilizing/destabilizing proteins or by affecting miRNA binding sites. In the *PTPRK* gene, one of the SNPs is located in a potential binding site for hsa-miR-1910 [24]. Furthermore, the CD SNP rs7559479 in the *IL18RAP* locus alters the binding efficiency of hsa-miR-140-3p, hsa-miR-212, and hsa-mir-27a, while another SNP in the same area (rs7603250) affects the binding of hsa-miR-668. It is important to note that rs7559479 also creates a potential binding site for has-miR-136 (as predicted by snpinfo.niehs.nih.gov). We consider it interesting that the *IL18RAP* gene displays the strongest e-QTL effect [6], corroborating the hypothesis that miRNAs may affect the expression of *IL18RAP*. Moreover, some of the CD-risk “top-SNPs” (i.e., the SNPs with the lowest *P* values) show overlap with genes encoding non-coding RNAs (ncRNAs), such as microRNAs (miRNAs), long intergenic non-coding RNAs (lincRNAs), or small nucleolar RNAs (snoRNAs) (unpublished results), indicating that additional layers of gene regulation and gene-splicing are involved in the disease mechanism. This finding should not be surprising as about 16 % of the loci associated with complex diseases do not harbor protein-coding genes [43, 44]. Altogether, it has become clear that ~95 % of the CD-risk SNPs are located in regulatory regions (Fig. 3a). The fine mapping of CD loci is ongoing and more light will be shed on the role of these regions in the etiology of CD.

## Expression QTL analysis can help to identify the causative gene in a locus with multiple candidates

It is difficult to identify the causal gene in a disease-associated locus that contains multiple candidate genes. The fact that the disease-associated SNP may not be the causal SNP, in strong LD with the true causal variant, it adds to the problem of identifying the causal gene. An elegant strategy that can be applied to narrow down the causal gene in a locus is to correlate genotypes with expression data. This approach has been coined expression QTL analysis [45–47]. Although eQTL analysis does not prove that the gene is the causal one in the locus, it can help in prioritizing genes for follow-up studies.

eQTLs come in two flavors: (1) *cis*-eQTLs in which SNPs affect expression of nearby genes [48], and (2) *trans*-eQTLs in which SNPs affect the expression of genes far away on the same chromosome or even on another chromosome [48]. Dubois et al. [23] used a dataset consisting of genome-wide gene expression data and genome-wide SNP data of 1,469 human primary blood leukocytes to perform an eQTL analysis in CD. They showed that 20 out of the 38 (53 %) non-HLA CD susceptibility loci they investigated displayed significant eQTL effects. The most impressive eQTL effect was found for SNP rs917997 in the *IL18RAP* gene (*P* = 7.4 × 10e − 87) causing a 9-fold difference of *IL18RAP* expression between carriers of two wild-type alleles versus carriers of two risk alleles [6]. This helped to pinpoint *IL18RAP* as the likely causal gene in a locus also harboring *IL18R1*, *IL1RL1*, and *IL1RL2,* since the latter three did not display a *cis*-eQTL effect. Altogether these findings indicate that the mechanism underlying CD is governed by a deregulation of gene expression. Other immune-related diseases show similar numbers of eQTLs for disease-associated SNPs, suggesting that this is a more general phenomenon [6]: for example, 39 out of 71 CrD loci (55 %) show an eQTL effect [49], and 32 out of 53 T1D loci (60 %) [15].

The identification of eQTL effects in *trans* (*trans*-eQTLs) is much more difficult, presumably since these are more tissue specific and cell specific [50]. *Trans*-eQTLs are of interest because they implicate biological processes by linking disease SNPs to the expression pattern of many genes, thereby potentially revealing disease-associated pathways. As an example, Fehrmann et al. [48] described the *trans*-eQTL effects of 1,167 published trait- or disease-related SNPs on gene expression in peripheral blood mononuclear cells (PBMCs) of 1,469 unrelated individuals. T*rans*-eQTL effects were observed on 113 genes, of which 46 could be replicated in a dataset obtained from monocytes of 1,490 different individuals, and 18 could be replicated in a dataset generated from subcutaneous adipose, visceral adipose, liver and muscle tissue from the same replication cohort. In addition, they identified 18 unlinked SNP pairs, associated with a single phenotype and affecting the regulation of the same *trans*-gene. The fact that singular genes are regulated in *trans* by multiple SNPs could indicate the importance of the *trans-*gene in the disease mechanism. In the same study, they also found that HLA SNPs are 10-fold enriched for *trans*-eQTL effects [48].

## Applying pathway analysis to zoom in on gene function and disease mechanisms

Although the GWAS approach has its shortcomings, for instance it cannot pinpoint the causal gene in all loci, the approaches described above can help suggest causal candidate genes. A significant subset of the CD susceptibility loci can be associated with T cell biology, including *REL, TNFAIP3, THEMIS/PTPRK, ETS1, RUNX3, TLR7/TLR8, BACH2,* and *IRF4* [19], but it is likely that other cell types are affected as well. Yet another strategy that can be applied to GWAS results is pathway analysis and quite a number of pathway analysis tools are now publicly available [51, 52]. In some of the pathway analysis approaches, human datasets have successfully been intersected with results obtained from model organisms such as yeast, worms and flies, to infer functional and physical interaction networks [53]. Pathway analysis algorithms predict pathways based on connections between the genes in the query list that can be distilled from literature co-citation, gene ontology terms, co-expression, protein-protein interaction data, possession of common regulatory motifs or domains, tissue-specific co-expression, subcellular co-localization, and phenotypic profiling. All of these sources of information have been shown to provide useful data on biological function. Using these data and insights, systems biology approaches [54] can then be applied to unravel the role of the immune system in CD. While these approaches have so far been less often applied in mammalian systems, the recent availability of relevant datasets in humans and mice will facilitate such strategies.

In a recent review Wang et al. outlined the development of pathway-based approaches for GWAS and discussed their practical use and caveats [51]. Many of the available tools examine whether a group of related genes in the same functional pathway are jointly associated with a trait of interest. Gene Relationships Among Implicated Loci (GRAIL) is a computational tool that takes a list of GWAS regions and predicts the likely causal gene in each locus using information from 250,000 PubMed abstracts [55]. GRAIL can predict new loci and was successfully applied to RA, where it identified CD28, PRDM1, and CD2/CD58 as involved in the disease [56]. Functional relationships between genes and their products can also be obtained from the Kyoto Encyclopedia of Genes and Genomes [57], the Biomolecular Interaction Network Database [58], the Human Protein Reference Database [59], the Gene Ontology (GO) Database [60], predicted (tissue-specific) phenome-interactome/expression networks [61, 62], the CCSB Interactome Database [63], and microarray co-expression datasets (GEMMA; http://www.chibi.ubc.ca/Gemma).

**Fig. 4:**
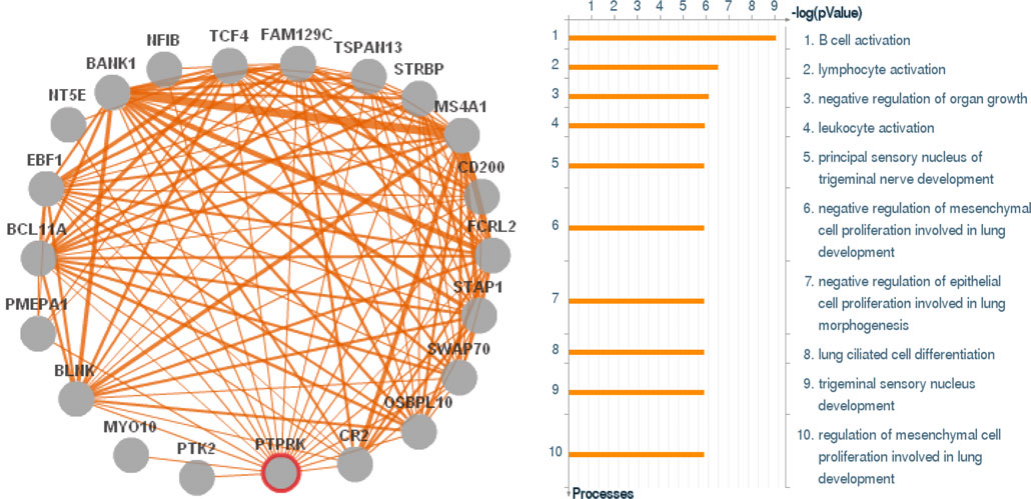
Co-expression analysis to predict the function of *PTPRK* gene. The *left panel* lists the genes showing co-expression with *PTPRK* in at least 15 different microarray datasets (extracted from the GEMMA co-expression database) and depicts the presence of interactions between those genes. The *width of the lines* represents the number of datasets (ranging from 15 to 25) containing evidence for the interaction. The *right panel* displays the results of an enrichment analysis performed on the *PTPRK* co-expressed genes, using the MetaCore GeneGo tool (see text). The *x*-axis displays significance for each of the biological processes plotted on the *y*-axis

Functionally related genes tend to be co-regulated transcriptionally, although the regulatory mechanisms can be extremely complex [64]. Despite this complexity, it is feasible to predict the function of a gene based on its “co-expressed gene signature”. As an example, GEMMA was used to acquire the gene set that is co-expressed with PTPRK, a gene with an unknown function. Subsequently, a commercially available pathway analysis suite—MetaCore-GeneGO (www.genego.com/metacore.php)—was used to search for significant enrichment terms to suggest a function for PTPRK. The enrichment analysis suggested that PTPRK is involved in B cell activation (Fig. 4). Although this observation has not yet been followed up, this illustrates that these kinds of approaches can be readily applied to generate novel hypotheses.

**Fig. 5:**
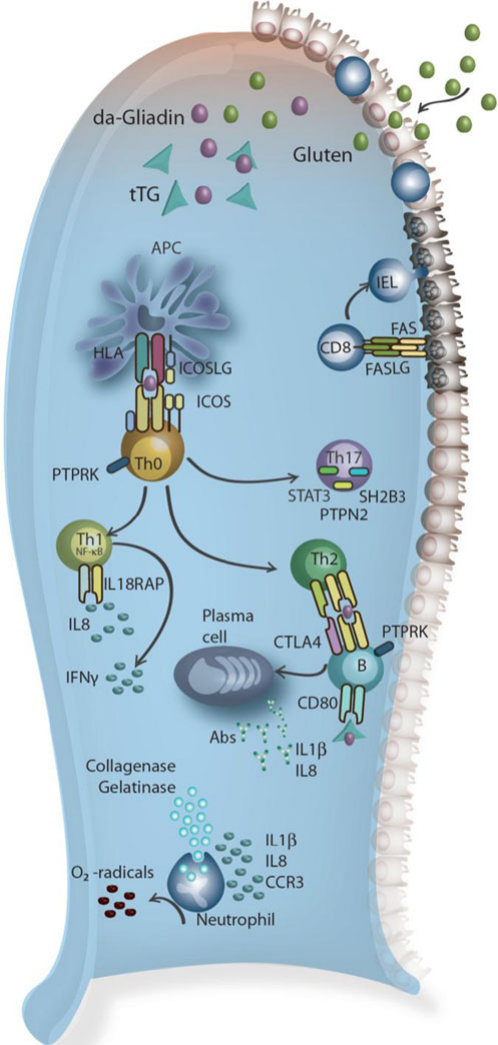
Immune cell types implied to be involved in celiac disease by pathway analyses. Gluten molecules, the environmental trigger of CD, are degraded into gliadins which in turn are modified by tissue transglutaminase (*tTG*) into deamidated gliadin (*da-Gliadin*). The latter peptides are presented to the immune system, resulting in activation of various immune cell types (according to pathway analyses, see text). For a more detailed description of the genes involved in these processes, see the text and reviews by Trynka et al. [14] and Abadie et al. [67]. *Abs*, antibodies; *FASLG*, *FAS* ligand; *ICOSLG*, *ICOS* ligand; *IEL*, intra-epithelial lymphocytes

When performing pathway analyses it is important to identify the correct tissue or cell type in which the disease gene probably operates [65]. For this, public databases such as BioGPS [66] can be used. The generally accepted view on CD pathogenesis is that CD is a T cell-mediated enteropathy in which T cells are major players in recognizing gluten epitopes in the context of HLA alleles and inducing anti-gluten T cell responses [67]. However, a BioGPS analysis using a human expression dataset [68] associates the CD loci not only with T cells (*TAGAP, TNFRSF14, CCR4, CTLA4, UBASH3A,* and *CD28*), but also with NK cells (*UBE2E3, RUNX3, FASLG, PTPN2,* and *IL18RAP*), neutrophils (*PLEK* and *CCR3*), and B cells (*BACH2, SOCS1, POU2AF1, ICOSLG, IRF4, CIITA, ZFP36L1,* and *CSK*) (Fig. 5). The results indicate that this tissue- or cell-specific approach can assist in generating new biological hypotheses. The BioGPS results suggest a role for NK cells in CD, while it has previously been shown that an impaired distribution of intraepithelial NK cells induces permanent loss of tolerance to gliadin [69] and that a deficiency of NK cells is involved in CD [70]. On the other hand, it is possible that the affected “NK cell genes” do not affect NK cell function, but that they are involved in the pathology mediated by intraepithelial lymphocytes (IELs) associated with CD, as it has been reported that CD IELs are derived from CD8 T cells but that they start expressing NK cell effector molecules [67]. Neutrophils may cause impaired intestinal barrier function by inducing a chronic inflammation and could thereby contribute to CD pathogenesis [71]. Lastly, B cells could be involved in presenting gluten to T cell receptors and thus contribute to the amplification of the anti-gluten T cell response [72]. Altogether these results suggest that this kind of pathway analysis yields clinically relevant information about the contribution of multiple immune cell types to CD pathology.

It has to be kept in mind that pathway analysis is based on the use of databases that contain experimental data and that the quality of this data is not equally high for every dataset included. Moreover, these tools favor the well-defined pathways [73] and lesser-studied genes may not be taken into account, making it more difficult to identify lesser known genes and pathways involved in disease etiology. Despite these shortcomings, pathway analysis approaches are becoming a mainstay in medical research and they have already demonstrated their usefulness in generating new hypotheses that can subsequently be tested.

## Conclusions

**Fig. 6:**
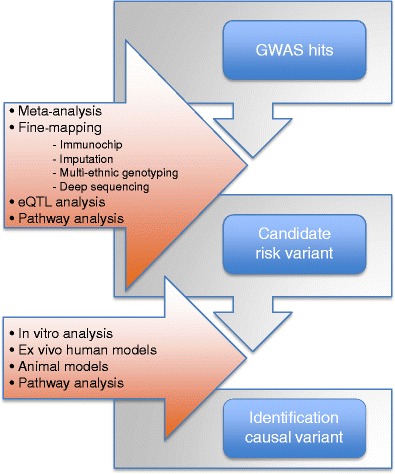
Summary of strategies to identify causal variants and disease mechanisms. GWAS association signals can be followed up by meta-analysis and/or fine-mapping to identify specific causal variants. Pathway and eQTL analyses can be applied to prioritize the causative genes and to generate hypotheses to explain the biological link between a causal gene and disease. Identified causal variants and genes can in turn be followed up by experiments by, for instance, ex vivo stimulation experiments using human or animal immune cells or by experiments with inflammation models in whole animals

Despite decades of research on CD, we still do not understand the exact mechanisms underlying this disease. However, the recent GWAS and follow-up studies have started to uncover the genetic components contributing to this disease. Although on the genetic level immune-related diseases still show differences in, for example, the number of disease susceptibility loci, the effect sizes associated to each locus, and the environmental factors involved in the various diseases [10], it is also clear that there is a remarkable overlap of susceptibility factors between various immune-related diseases [2, 15–22]. This overlap clearly implies the involvement of shared pathways in multiple autoimmune diseases and, most importantly, suggests that general treatment modalities might be feasible for some immune-related diseases. However, not all of the results obtained so far can be readily interpreted as the resolution of the SNP analyses is, in many cases, still not high enough. Many susceptibility loci—also shared loci—still contain multiple genes. Several strategies can be applied to pinpoint the causal variants in these loci (Fig. 6) and it can be expected that, in the near future, combinations of these approaches, which involve the integration of complex datasets containing different levels of information, will identify novel causal variants associated with immune-related diseases. The elucidation of these novel components has immediate clinical relevance, as they can be included in genetic-risk modeling approaches [74]. Moreover, they might represent novel biomarkers for celiac disease, enabling physicians to diagnose all at-risk patients, preferably before the onset of symptoms, which would greatly reduce the overall cost to society and the burden on patients. Most importantly, the causal variants, or other molecules that have been identified as playing a role in the same pathway, represent new potential therapeutic targets, not only for celiac disease but also other autoimmune diseases.
